# Comparison the effect of gelatin sponge and epinephrine-soaked gauze for hemostasis and pain control after hemorrhoidal surgery

**DOI:** 10.1038/s41598-023-45380-0

**Published:** 2023-10-21

**Authors:** Kun-Min Tsai, Kee-Thai Kiu, Min-Hsuan Yen, Yu-Chun Yen, Ka-Wai Tam, Tung-Cheng Chang

**Affiliations:** 1 Department of General Medicine, Shuang Ho Hospital, Taipei Medical University, No. 901, Zhonghua Road, Yongkang District, Tainan City, New Taipei City, 71004 Taiwan; 2 Division of Colorectal Surgery, Department of Surgery, Shuang Ho Hospital, Taipei Medical University, Number 291, Zhongzheng Road, Zhonghe District, New Taipei City 235, Taiwan; 3 Biostatistics Center, Office of Data Science, Taipei Medical University, No. 172-1, Sec. 2, Keelung Road, Taipei City 106, Taiwan; 4 Division of General Surgery, Department of Surgery, Taipei Medical University - Shuang Ho Hospital, Number 291, Zhongzheng Road, Zhonghe District, New Taipei City 235, Taiwan; 5 Division of General Surgery, Department of Surgery, School of Medicine, College of Medicine, Taipei Medical University, New Taipei City, Taiwan; 6 Cochrane Taiwan, Taipei Medical University, Taipei City, Taiwan; 7 Department of Surgery, School of Medicine, College of Medicine, Taipei Medical University, Taipei City, Taiwan

**Keywords:** Anal diseases, Outcomes research

## Abstract

Post-operative pain and bleeding are the main complications following hemorrhoidal surgery. This study aimed to investigate whether an absorbable gelatin sponge is a superior hemostatic and analgesic agent compared to gauze soaked in epinephrine for post-hemorrhoidal surgery care. A retrospective study was conducted using data from a single institute. Data were collected from the electronic medical record database and outpatient patient questionnaire archive. The study encompassed 143 patients who received gauze soaked in epinephrine as the hemostatic agent after hemorrhoidal surgery and 148 patients who received an absorbable gelatin sponge. Most patients underwent stapled hemorrhoidopexy, with 119 (83.2%) in epinephrine group and 118 (79.7%) in gelatin sponge group. The primary outcome measurements were postoperative pain score, oral analgesic dosage and complications. Patients in the absorbable gelatin sponge group reported significantly lower pain scores from 8 h after their hemorrhoidal surgery (postoperative day 0) through postoperative day 2. The average pain scores in the absorbable gelatin sponge group and gauze soaked in epinephrine group were 5.3 ± 3.2 and 6.2 ± 3.2 (*p* = 0.03) on postoperative 8 h; 4.7 ± 3.0 and 5.8 ± 2.9 (*p* ≤ 0.01) on postoperative day one; and 4.4 ± 2.8 and 5.3 ± 2.9 (*p* = 0.01) on postoperative day two, respectively. There were no significant differences in postoperative recovery or complication rates between the two groups. Our study revealed that absorbable gelatin sponges provide more effective pain relief to patients during the initial postoperative days after hemorrhoidal surgery, without any adverse impact on patient outcomes. Consequently, absorbable gelatin sponges are recommended as a replacement for gauze soaked in epinephrine following hemorrhoidal surgery.

## Introduction

For patients suffering from high-grade hemorrhoidal disease, surgical intervention is the prevailing course of action to alleviate symptoms^1,2^. Among the surgical options, Milligan–Morgan hemorrhoidectomy and stapled hemorrhoidopexy stand out as commonly employed procedures for addressing hemorrhoidal disease. Nonetheless, these surgical interventions may lead to postoperative discomfort and potential complications, including postoperative bleeding, urinary retention, or infection. In cases where wound healing is delayed, a patient's recovery and return to their regular daily activities can be protracted^3^.

Hemorrhoidectomy involves the excision the vascular cushions of the anus, a procedure inevitably associated with bleeding^3^. Consequently, determining the optimal method for achieving hemostasis after hemorrhoidectomy or stapled hemorrhoidopexy is a valuable research focus. One of the common methods of achieving hemostasis after hemorrhoidal surgery is the placement of gauze soaked in epinephrine in the patient’s anal canal. This soaked gauze induces vasoconstriction and applies pressure, effectively halting the bleeding. However, gauze is nonabsorbable and requires postoperative removal, which can lead to discomfort for patients. To alleviate patient discomfort, some surgeons may opt for alternative materials instead of gauze, with absorbable gelatin sponges being one such choice.

Absorbable gelatin sponge is a medical material intended for application to bleeding surfaces as a hemostatic agent. They are a water-insoluble, off-white, nonelastic, porous, and pliable product prepared from purified porcine skin. If an absorbable gelatin sponge is used as the hemostatic agent after hemorrhoidal surgery, it does not have to be removed postoperatively—this may reduce patient discomfort during postoperative recovery^4^.

Few studies have explored the utilization of absorbable gelatin sponges following hemorrhoidal surgery. Therefore, we conducted a retrospective study to compare the outcomes of employing an absorbable gelatin sponge with those of using gauze soaked in epinephrine after hemorrhoidal surgery. We anticipate that this study will yield valuable evidence to inform the choice of a hemostatic agent following hemorrhoidal surgery.

## Materials and methods

### Ethical information

This study was conducted in accordance with the Declaration of Helsinki and was approved by the Taipei Medical University Joint Institutional Review Board (TMU-JIRB; number: N202009024). Because this was a retrospective study, informed consent was not required by the Taipei Medical University Joint Institutional Review Board.

### Patients

This study enrolled patients who underwent hemorrhoidal surgery at Taipei Medical University Shuang-Ho Hospital between March 2020 and February 2021. Specifically, patients with grade III or grade IV hemorrhoidal disease were included in the study, while those with an anal fistula or rectal polyps, as well as those who underwent emergency hemorrhoidal surgery or combined surgery, were excluded. Patients who received an absorbable gelatin sponge as a hemostatic agent are in the gelatin sponge group, and patients who received gauze soaked in epinephrine are in the epinephrine group.

### Procedure

All patients were admitted to the hospital 1 day prior to their scheduled hemorrhoidal surgery. The choice of surgical procedure, either stapled hemorrhoidopexy or Milligan-Morgan hemorrhoidectomy, was determined through discussions between the surgeons and patients. In the case of patients undergoing stapled hemorrhoidopexy, the PROXIMATE PPH Circular Stapler, a 33-mm hemorrhoidal circular stapler, was utilized. The majority of patients underwent surgery in the jackknife position, with either spinal or general anesthesia administered. The anesthetics drug for spinal anesthesia is 0.5% bupivacaine hydrochloride. The surgical team adhered to uniform procedures for both hemorrhoidectomy and stapled hemorrhoidopexy.

### Hemostasis

At the end of the hemorrhoidal surgery, the surgeon placed an absorbable gelatin sponge (50 mm × 70 mm × 10 mm) or gauze soaked in epinephrine in the patient’s anal canal after complete hemostasis was achieved. In the gelatin sponge group, the sponge was inserted into the anal canal as the surgical wound dressing, and it would degrade naturally or be discharged during the patient's first defecation. In the epinephrine group, a single 4-in. by 4-in. gauze was soaked in a 1 mL solution containing 1 mg/mL of epinephrine, which was diluted with 10 mL of normal saline. This gauze, saturated with the epinephrine solution, was then inserted into the anal canal to function as the surgical wound dressing. The gauze with epinephrine were removed 8 h after the surgery by a nurse. To prevent potential adverse effects associated with the use of topical epinephrine, epinephrine-soaked gauze will not be employed as a hemostatic agent in the following situations: 1. patients undergoing anesthesia with halothane; 2. patients with a history of closed-angle glaucoma; 3. patients with catecholaminergic polymorphic ventricular tachycardia; 4. patients with hypersensitivity to sympathomimetic drugs^5^. In all cases, 2% Xylocaine jelly was applied as a lubricant during the insertion of both the gelatin sponge and the epinephrine-soaked gauze. This was done to prevent the risk of anal lacerations during the insertion and removing process.

### After hemorrhoidal surgery

After their hemorrhoidal surgery, patients were requested to rest in bed for 8 h. Subsequently, they were instructed to take a sitz bath four to six times per day for 10 min at a time. Patients without any postoperative complications were discharged on the day following their hemorrhoidal surgery. In postoperative pain management protocol, 40 mg of parecoxib was administered intravenously every 12 h during the first 24 h after surgery. Additionally, patients were also provided with oral 25 mg diclofenac or 500 mg acetaminophen, four times a day (QID), for a duration of 2 weeks following the surgery. The administration of analgesics was adjusted based on patient requests and individual needs. A visual analog scale (VAS) score was used for measuring pain, with 0 representing no pain and 10 representing the worst pain ever experienced.

Patients were instructed to maintain a daily record of their pain scores, oral analgesic dosages, and the frequency of defecation as a standard component of our routine follow-up procedure. Additionally, outpatient department follow-up appointments were scheduled for patients at both 1 week and 1 month after their discharge from the hospital. During these outpatient department follow-ups, the surgeon conducted examinations of the patients' surgical wounds and reviewed their medical records. This comprehensive review allowed for the determination of the total number of analgesic drug doses taken, assessment of pain scores, and identification of any postoperative complications that may have arisen.

### Definition of complications

Postoperative bleeding was defined as bleeding that required surgical intervention or that caused the patient to return to the hospital within 14 days after hemorrhoidal surgery. Urinary retention was defined as a patient requiring urinary catheterization within 14 days after hemorrhoidal surgery. Local infection was defined as a patient developing a perianal abscess within 14 days after hemorrhoidal surgery.

### Statistical analysis

Continuous variables were presented as mean and standard deviation, and categorical variables were presented as number and percentage of the category. A two-sample *t* test was used to compare continuous variables, and a chi-squared test or Fisher’s exact test was used to compare categorical variables. All statistical tests were two-tailed, and a *p* value less than 0.05 indicated statistical significance. A generalized estimating equation was used to analyze other possible factors associated with differences in patients’ postoperative course.

## Results

Two hundred ninety-one patients underwent hemorrhoidal surgery in a single institute from March 2020 to February 2021. However, three of these patients were excluded from the study because two patients had undergone emergency hemorrhoidectomy and the other had undergone hemorrhoidectomy under local anesthesia. Consequently, the analysis included a total of 291 patients. The surgical records revealed that 143 of these patients received gauze soaked in epinephrine, constituting the epinephrine group, while the remaining 148 patients received an absorbable gelatin sponge, forming the absorbable gelatin sponge group.

### Patient characteristics

The study population ranged in age between 20 and 82 years and had no significant differences in mean age, gender, hemorrhoid grade, body mass index, American Society of Anesthesiologists (ASA) physical status classification or surgery type. In the epinephrine group, 72 patients were men and 71 were women, and in the gelatin sponge group, 58 were men and 90 were women. Among the patients in the epinephrine group, there were 42 individuals with grade III hemorrhoids and 101 with grade IV hemorrhoids. In gelatin sponge group had 31 patients with grade III hemorrhoids and 117 with grade IV. The majority of patients received spinal anesthesia, with only 5 patients undergoing general anesthesia. In terms of the type of surgery performed, the majority of patients in both groups underwent stapled hemorrhoidopexy, with 119 patients (83.2%) in the epinephrine group and 118 patients (79.7%) in the gelatin sponge group. Further details regarding patient characteristics can be found in Table 1.

**Table 1:** Characteristics of all patients.

	Epinephrine		Gelatin sponge		*p* value
	*n* = 143		*n* = 148		
Age (years), mean (SD)	48.8	(14.0)	47.3	(12.22)	0.33^a^
Gender					0.06^b^
Male	72	(50.3%)	58	(39.1%)	
Female	71	(49.7%)	90	(60.9%)	
BMI (kg/m^2^), mean (SD)	24.4	(4.1)	24.1	(4.4)	0.66^a^
Diabetes mellitus	8	(5.6%)	10	(6.8%)	0.68^b^
Hypertension	23	(16.1%)	29	(19.6%)	0.44^b^
Symptom					
Bleeding	77	(53.8%)	75	(50.7%)	0.55^b^
Prolapse	104	(72.7%)	119	(80.4%)	0.15^b^
Pain	70	(49.0%)	75	(50.7%)	0.82^b^
Duration of symptom					0.99^b^
< 1 month	27	(18.9%)	26	(17.6%)	
1–12 months	24	(16.8%)	31	(20.9%)	
> 12 months	92	(64.3%)	91	(61.5%)	
Hemorrhoid grade					0.10^b^
III	42	(29.4%)	31	(20.9%)	
IV	101	(70.6%)	117	(79.1%)	
Previous surgery					0.40^b^
Yes	8	(5.6%)	12	(8.1%)	
ASA classification					0.19^c^
I	63	(44.1%)	79	(53.4%)	
II	80	(55.9%)	67	(45.2%)	
III			2	(1.4%)	
Anesthesia type					0.563^b^
General	3	(2.1%)	2	(1.4%)	
Spinal	140	(97.9%)	146	(98.6%)	
Surgery type					0.45^b^
Milligan–Morgan hemorrhoidectomy	24	(16.8%)	30	(20.3%)	
Stapled hemorrhoidopexy	119	(83.2%)	118	(79.7%)	
Surgery duration (min), mean (SD)	13.6	(6.2)	12.6	(4.8)	0.13^a^
40 mg parecoxib administration times	1.8	(1.3)	1.7	(1.3)	0.72^a^

*BMI* body mass index, *ASA score* the American Society of Anesthesiologists physical status classification.

^a^*t* test.

^b^Chi-square test.

^c^Fisher’s exact test.

### Subgroup analysis of stapled hemorrhoidopexy and hemorrhoidectomy

In stapled hemorrhoidopexy, there were 119 patients in the epinephrine group and 118 patients in the gelatin sponge group. In hemorrhoidectomy, there were 24 patients in the epinephrine group and 30 patients in the gelatin sponge group. No significant differences were observed in terms of mean age, gender distribution, hemorrhoid grade, body mass index, ASA physical status classification, or symptom duration between the epinephrine group and the gelatin sponge group, whether the patients underwent stapled hemorrhoidopexy or hemorrhoidectomy. For more detailed patient characteristics, please refer to Table 2.

**Table 2:** Subgroup analysis of patients with stapled hemorrhoidopexy and hemorhoidectomy.

Baseline characteristics	Stapled hemorrhoidopexy				*P* value	Milligan-Morgan Hemorrhoidectomy				*P* value
	Epinephrine	n = 119	Gelatin sponge	n = 118		Epinephrine	n = 24	Gelatin sponge	n = 30	
Age, mean (SD)	48.9	(13.1)	47.0	(11.6)	0.25^a^	48.6	(18.0)	48.6	(14.2)	0.99^a^
Sex, n					0.06^b^					0.55^b^
Men	58	(48.7%)	43	(36.4%)		14	(58.3%)	15	(50%)	
Women	61	(51.3%)	75	(63.6%)		10	(41.7%)	15	(50%)	
BMI, mean (SD)	24.4	(4.29)	24.2	(4.5)	0.68^a^	24.0	(3.4)	23.9	(4.2)	0.92^a^
HTN, n (%)	18	(15.1%)	22	(18.6%)	0.47^b^	5	(20.8%)	7	(23.3%)	0.83^b^
DM, n (%)	5	(4.2%)	8	(6.8%)	0.39^b^	3	(12.5%)	2	(6.7%)	0.47^b^
Hemorrhoid grade, n (%)					0.06^b^					0.70^b^
III	37	(31.1%)	26	(22.0%)		5	(20.8%)	5	(16.7%)	
IV	82	(68.9%)	92	(78.0%)		19	(79.2%)	25	(83.3%)	
Symptoms, n (%)										
Bleeding	67	(56.3%)	57	(48.3%)	0.19^b^	10	(41.7%)	18	(60%)	0.19^b^
Prolapse	90	(75.6%)	98	(83.1%)	0.20^b^	14	(58.3%)	21	(70%)	0.38^b^
Pain	55	(46.2%)	54	(45.8%)	0.90^b^	15	(62.5%)	21	(70%)	0.57^b^
Duration, n (%)					0.94^c^					0.98^c^
< 1 month	21	(17.6%)	18	(15.3%)		6	(25%)	8	(26.7%)	
1–12 months	19	(16.0%)	26	(13.6%)		5	(20.8%)	5	(16.7%)	
> 12 months	79	(66.4%)	74	(62.7%)		13	(54.2%)	17	(56.7%)	
Previous treatment, n (%)	6	(5.0%)	8	(6.8%)	0.57^b^	2	(8.3%)	4	(14.81%)	0.57^b^
ASA score, n					0.14^c^					0.91^c^
I	53	(44.5%)	65	(55.1%)		10	(41.7%)	14	(46.7%)	
II	66	(55.5%)	52	(44.1%)		14	(58.3%)	15	(50.%)	
III			1	(0.8%)				1	(3.3%)	
Anesthesia, n (%)					0.66^b^					0.99^b^
General	2	(1.7%)	2	(1.7%)						
Spinal	117	(98.3%)	116	(98.3%)		24	(100%)	30	(100%)	
40 mg parecoxib administration times	1.8	(1.2)	1.8	(1.3)	0.78	1.7	(1.6)	1.6	(1.4)	0.87^a^

*BMI* body mass index, *DM* diabetes mellitus, *HTN* hypertension, *QID* quarter in die, *SD* standard deviation.

^a^*t* test.

^b^Chi-square test.

^c^Fisher exact test.

### Postoperative pain

**Figure 1:**
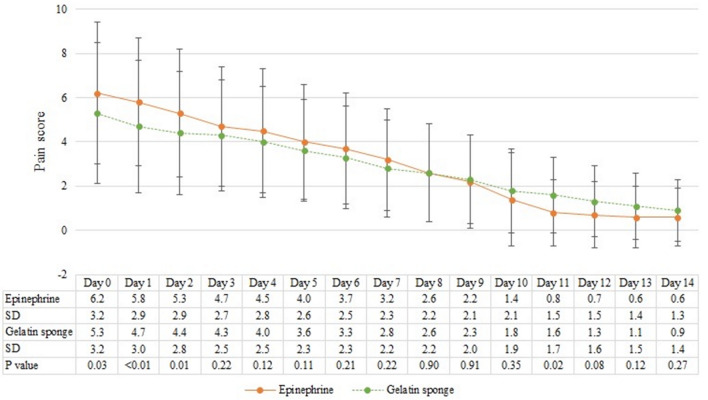
Daily postoperative pain score. The continuous progression of the pain score in days, with mean and standard deviation. The Y-axis represents the pain score, where 0 represents no pain and 10 represents the worst pain ever experienced. *SD* standard deviation.

Patients were instructed to document their daily pain scores starting from 8 h after their hemorrhoidal surgery and continuing until postoperative day 14 as part of our routine follow-up. The average pain scores of the absorbable gelatin sponge group and the gauze soaked in epinephrine group were 5.3 ± 3.2 and 6.2 ± 3.2 (*p* = 0.03) at postoperative 8 h; 4.7 ± 3.0 and 5.8 ± 2.9 (*p* < 0.01) on postoperative day 1, respectively; and 4.4 ± 2.8 and 5.3 ± 2.9 (*p* = 0.01) on postoperative day 2, respectively. The results indicate that the absorbable gelatin sponge group consistently reported significantly lower pain scores on postoperative 8 h, day 1, and day 2 compared to the gauze soaked in epinephrine group (Fig. 1).

**Figure 2:**
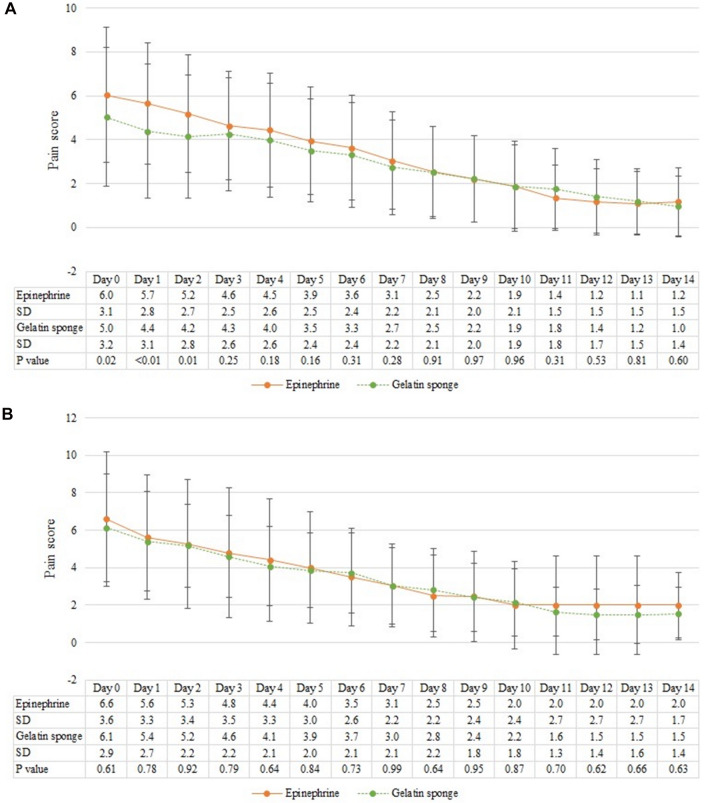
Daily postoperative pain score of patients underwent (**A**) stapled hemorrhoidopexy and (**B**) hemorrhoidectomy. The continuous progression of pain score in days with mean and standard deviation. Y-axis stands for pain score where 0 represented no pain and 10 represented the worst pain ever experienced. *SD* standard deviation.

The continuous progression of postoperative pain scores in patients who underwent stapled hemorrhoidopexy is depicted in Fig. 2A. Notably, the pain scores were significantly higher in the epinephrine group compared to the gelatin sponge group during the initial 3 days following the procedure. Specifically, at postoperative 8 h, the mean pain scores for both groups were 6.0 ± 3.1 and 5.0 ± 3.2, respectively (*p* = 0.02). On postoperative day 1, the scores were 5.7 ± 2.8 and 4.4 ± 3.1, respectively (*p* < 0.01), and on postoperative day 2, they were 5.2 ± 2.7 and 4.2 ± 2.8, respectively (*p* = 0.01). In contrast, as illustrated in Fig. 2B, no significant difference in postoperative pain scores was observed between the epinephrine group and the gelatin sponge group among patients who underwent hemorrhoidectomy.

### Oral analgesics dosage

**Figure 3:**
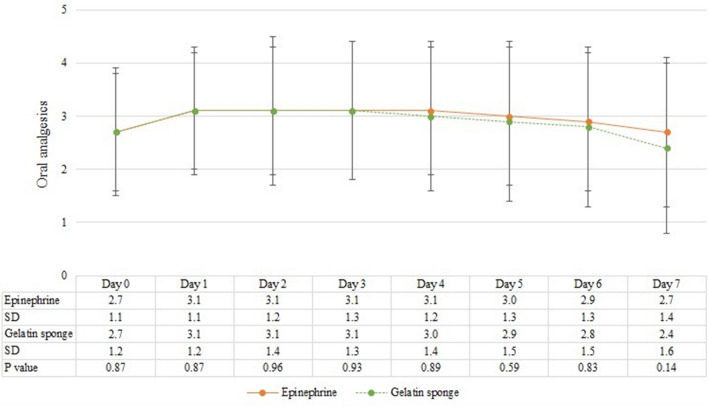
Daily consumption of oral analgesics in days, with mean and standard deviation. The Y-axis represents the number of acetaminophen or diclofenac tablets patients consumed each day. *SD* standard deviation.

**Figure 4:**
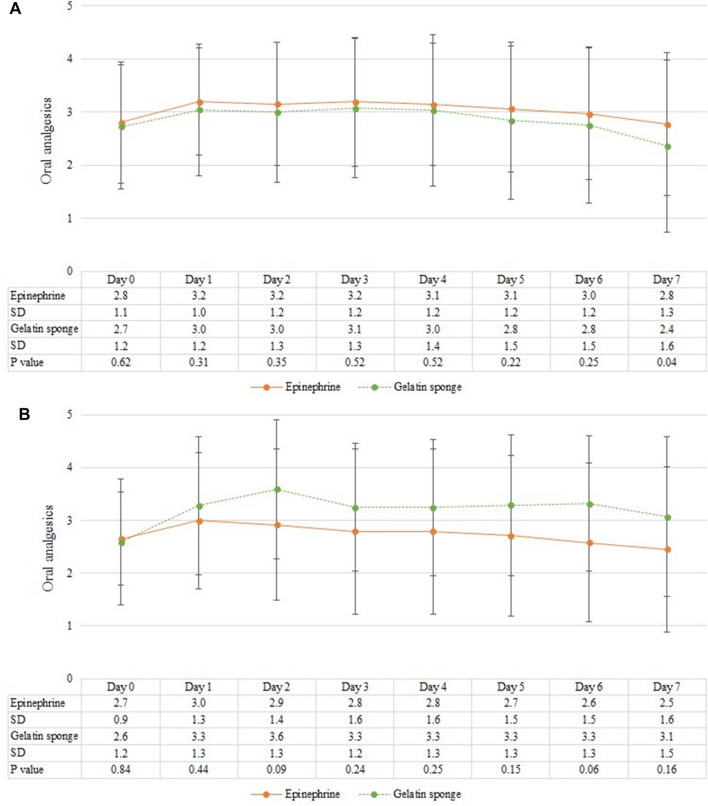
Daily consumption of oral analgesics by patients who underwent (**A**) stapled hemorrhoidopexy and (**B**) hemorrhoidectomy. The data includes the mean daily tablet consumption, represented with error bars indicating the standard deviation. The Y-axis quantifies the daily amount of analgesics tablets consumed by patients. *SD* standard deviation.

Patients were requested to record their daily oral analgesic dosage from postoperative 8 h to postoperative day 7. No significant difference was identified between the two groups (Fig. 3). In subgroup analysis, there was no significant difference observed in analgesic consumption between the two groups in the case of stapled hemorrhoidopexy (Fig. 4A) or in conventional hemorrhoidectomy (Fig. 4B).

### Other postoperative outcomes

There were no statistically significant differences observed between the two groups in terms of the following variables: length of hospital stays, postoperative complications including bleeding, urinary retention, or local infection, the number of patients requiring additional medical management due to complications within 14 days of surgery, the duration until the first postoperative defecation, or the time it took for patients to return to work (Table 3).

**Table 3:** Postoperative outcomes and complications.

	Epinephrine		Gelatin sponge		*p* value
	*n* = 143		*n* = 148		
Days of hospital stay, mean (SD)	2.3	(0.6)	2.3	(0.6)	0.81^a^
Complications					
Bleeding	2	(1.4%)	3	(2.0%)	0.68^b^
Urinary retention	2	(1.4%)	2	(1.4%)	0.97^b^
Local infection	1	(0.7%)	0	(0%)	0.31^b^
Additional management within 14 days*	4	(2.8%)	3	(2.0%)	0.67^b^
Days until first defecation, mean (SD)	1.4	(1.3)	1.3	(1.4)	0.31^a^
Days until go back to work, mean (SD)	5.8	(3.8)	5.5	(4.0)	0.55^a^

^a^*t* test.

^b^Fisher’s exact test.

*Additional management included patients received an operation, returned to the emergency department, or returned to the outpatient department due to complications.

## Discussion

Several previous studies have investigated the efficacy of absorbable gelatin sponges in achieving hemostasis in various surgical procedures^3,6,7^. However, the application of absorbable gelatin sponges in hemorrhoidal surgery has remained relatively unexplored. Therefore, this study aimed to compare the use of absorbable gelatin sponges with the utilization of gauze soaked in epinephrine among patients undergoing hemorrhoidal surgery. The findings of our study indicate that there was no significant difference in the complication rate between the two groups. Additionally, in terms of postoperative pain recovery, it was observed that the gelatin sponge group consistently reported significantly lower pain scores than the epinephrine group from 8 h postoperatively through to postoperative day 2.

Post-operative bleeding following anal surgery typically occurs within the first 24 h post-surgery, with an incidence rate ranging from 1.5 to 15.6% ^8–12^. Various methods are employed to ensure effective hemostasis at the conclusion of these procedures. One of the most commonly used hemostatic methods involves applying pressure within the anal canal to prevent bleeding^6^. This can be achieved using commercially available tamponed devices, such as polyvinyl alcohol, or by directly applying rolled gauze within the anal canal.

Few studies have been conducted to evaluate the post-operative outcomes of tamponade dressing after hemorrhoidal surgery. In a randomized controlled trial by Langenbach et al.^13^, patients with tamponade dressing experienced significant postoperative pain compared to those without tamponade dressing. Langenbach et al. also conducted a large-sample study comparing tamponade dressing and no dressing after hemorrhoidal surgery. This study revealed that post-operative pain scores were lower in the no dressing group during the first 6–12 h post-surgery than in the tamponade dressing group; however, the use of analgesics was similar in both groups^14^. In this study, the reduction in pain but not in the use of analgesics may be attributed to the fact that the difference in pain intensity between the two groups was only one point (from 6 to 5), which did not exceed the minimal clinically important difference^15,16^. Similar results were observed in our study. The gelatin used in this study is soft and water-soluble, which means it does not exert pressure on the anal canal as compared to gauze soaked with epinephrine. This reduction in pressure contributes to reduced post-operative pain in the first 2 days following hemorrhoidal surgery. However, the use of analgesics in the gelatin sponge group was relatively low, but it did not reach statistical significance. This may also be caused by the difference in pain between the two groups during the first 2 days after surgery in our study was about one point. Despite the absence of a significant difference in the use of analgesics between the two groups, the cost of gelatin sponge is approximately USD 6–7. Therefore, the use of gelatin sponge after hemorrhoid surgery to reduce patients' pain without significantly increasing hospitalization costs is a worthwhile endeavor.

Previous studies have indicated that hemorrhoidal surgery tends to result in more pronounced pain compared to other surgical procedures^17^. Consequently, numerous studies have been conducted to explore methods for alleviating postoperative pain following hemorrhoidal surgery^18–20^. In our current study, we found that absorbable gelatin sponges provided significantly superior pain relief for patients from postoperative 8 h to postoperative day 2 in comparison to gauze soaked in epinephrine. Our deduction is that absorbable gelatin sponges, being soluble and soft in nature, are less prone to adhering to wounds and tissue linings, thereby causing less pain due to the absence of foreign object compression. Additionally, the advantage of absorbable gelatin sponges lies in their ability to be naturally excreted during bowel movements, eliminating the need for invasive removal, which can be a source of pain, unlike gauze. Similar studies have shown that absorbable gelatin sponges can effectively reduce pain in patients undergoing septoplasty for reasons similar to those identified in our study^6^.

Our study did not reveal a significant difference in the incidence of postoperative hemorrhage between patients who received an absorbable gelatin sponge and those who were treated with gauze soaked in epinephrine. It's worth noting that in a previous study comparing the use of an absorbable gelatin sponge versus a non-sponge insert after transrectal prostate needle biopsy, the application of an absorbable gelatin sponge reduced the likelihood of rectal bleeding when compared to the use of a non-sponge insert^3^. However, there are potential reasons for the disparity between the results of that study and our own findings, including differences in how the hemostatic agent was applied and variations in surgical methods. Previous studies have highlighted that the primary factor influencing the rate of postoperative hemorrhage following hemorrhoidal surgery is the specific surgical technique employed^2^. However, it's important to note that other studies have suggested that the method of surgery may have minimal to no significant impact on the incidence of postoperative hemorrhage^2,21^.

Another common complication, urinary retention, did not exhibit a significant difference between the two groups in this study. Researchers have previously elucidated that urinary retention can have multiple underlying causes, such as irritation or obstruction of pelvic nerves and reflexes triggered by pain^2^. Importantly, these causes are not directly related to the choice of hemostatic substance used after surgery. In support of this, findings from another study focused on hemorrhoidectomy also indicated that the surgical method employed did not exert a substantial impact on the occurrence of urinary retention^11^.

The study has several limitations. Firstly, the retrospective nature of the study resulted in the loss of some data, particularly pain scores during postoperative follow-up. This data loss became more pronounced starting from postoperative day 7. Additionally, since the majority of patients underwent stapled hemorrhoidopexy, only 54 patients underwent hemorrhoidectomy in analysis. While patients in the epinephrine group exhibited higher pain scores on postoperative day 0 and day 1 in Fig. 2B, this difference did not reach statistical significance. Consequently, it may be necessary to increase the sample size for future research endeavors. Lastly, it was challenging to verify whether patients adhered to their prescribed pain relievers or if those with complications received subsequent care and treatment at our hospital. The data loss restricted our ability to track postoperative outcomes over the long term.

## Conclusion

In conclusion, this study has shown that utilizing an absorbable gelatin sponge as the hemostatic agent during the initial days following hemorrhoidal surgery provides superior pain relief compared to using gauze soaked in epinephrine. Furthermore, our findings did not reveal any indications of adverse effects associated with the use of an absorbable gelatin sponge. Consequently, we recommend the adoption of absorbable gelatin sponges as a suitable substitute for gauze soaked in epinephrine as the preferred hemostatic and compressive material in hemorrhoidal surgery.

## Data Availability

The datasets used for this study are available from the corresponding author on reasonable request.
